# Effects of cholinergic antagonists on ghrelin release and expression in the crop, proventriculus, and duodenum of newly hatched chickens

**DOI:** 10.3389/fphys.2025.1553474

**Published:** 2025-05-02

**Authors:** Colin Guy Scanes, Klaudia Jaszcza, Krystyna Pierzchala-Koziec

**Affiliations:** ^1^ Department of Biological Sciences, University of Wisconsin–Milwaukee, Milwaukee, WI, United States; ^2^ Department of Animal Physiology and Endocrinology, University of Agriculture in Krakow, Krakow, Poland

**Keywords:** ghrelin release, cholinergic receptors, ghrelin expression, ghrelin O-acyltransferase expression, growth hormone secretagogue receptor-1 expression, chicken

## Abstract

The aim of this study was to evaluate the effect of the cholinergic receptor antagonists on ghrelin (GHRL) expression and release from parts of the gastrointestinal system in an unique animal model—newly hatched chickens. Ghrelin was released from explants of the crop, proventriculus, and duodenum tissues *in vitro*. The expression of GHRL, along with that of ghrelin O-acyltransferase (GOAT) and growth hormone secretagogue receptor (GHSR-1a), was also observed in the crop, proventriculus, and duodenum. This is the first report on ghrelin expression, synthesis, and release in the avian crop. The release and expression of ghrelin, together with ghrelin-related parameters (expression of GOAT and GHSR-1a), were influenced by incubation with cholinergic antagonists, particularly in gastrointestinal explants from chicks within 2 h of hatching. For instance, there was increased release of ghrelin from crop or proventriculus explants from newly hatched chicks in the presence of hexamethonium. In addition, the expressions of ghrelin, GOAT, and GHSR-1a were increased in the presence of hexamethonium in crop explants from newly hatched chicks. In contrast, the release of ghrelin from duodenal explants was decreased in the presence of either atropine or hexamethonium in both newly hatched and 1-day-old chicks. There were relationships between ghrelin release and expression and also with GOAT and GHSR-1a expression, particularly in crop explants from newly hatched chicks. For instance, there were strong relationships (adjusted R2 > 0.84) between the expression of ghrelin, GOAT, and GHSR in tissue incubated with cholinergic antagonists. This is a novel report demonstrating ghrelin release and synthesis from three regions of the avian gastrointestinal tract. It also demonstrates the cholinergic control of ghrelin release and synthesis.

## 1 Introduction

Ghrelin (GHRL) is an important hormone that controls the secretion of growth hormones, feeding, and gastrointestinal functioning (Kojima et al., 1999). The major source of ghrelin is the stomach (humans, Ariyasu et al., 2001), but it is also present in the small intestine and colon (Date et al., 2000). Ghrelin has been identified in both the avian proventriculus and duodenum (Kaiya et al., 2002; Wada et al., 2003; Neglia et al., 2005). Cholinergic control of ghrelin release is established in mammals (rats, Hosoda and Kangawa, 2008; humans, Broglio et al., 2004; Maier et al., 2004), but it remains unknown whether such a system exists in birds. Moreover, there are unique structures in the gastrointestinal tract in birds that are not found in mammals; these are the proventriculus (equivalent to the glandular stomach) and the crop (an out-pocketing of the esophagus). It was hypothesized that ghrelin would be synthesized and released from both the duodenum and proventriculus and that this process would be under cholinergic control. In view of the presumptive absence of the synthesis of gastrointestinal hormones in the mammalian esophagus, it was considered unlikely that the crop would synthesize ghrelin. For completeness, cholinergic antagonists were included in the study examining ghrelin release and the expressions of *GHRL*, ghrelin O-acyltransferase (*GOAT*), and growth hormone secretagogue receptor (*GHSR-1a*). There is no published evidence for the release or expression of *GHRL* from the crop nor for the expression of *GOAT*. However, ghrelin influences the contractions of crop smooth muscle in chickens (Kitazawa et al., 2007). Moreover, its receptor, *GHSR-1a*, has been identified in the muscle layer of the chicken crop, together with both the smooth muscle and enteric neurons in the proventriculus (Kitazawa et al., 2007). Interestingly, the mode of action of ghrelin differs between the chicken proventriculus and crop, with ghrelin acting on both smooth muscle and enteric neurons in the proventriculus but only on the smooth muscle in the crop (Kitazawa et al., 2007).

Acetylcholine plays multiple roles in the gastrointestinal tract. For instance, the muscarinic antagonist atropine delayed mouth-to-ileum transition time in humans (Borody et al., 1985). The cholinergic agonist carbachol induces contractions in the ileum (Caputi et al., 2017). In addition, the muscarinic cholinergic antagonist atropine blocks the increase in gastric acid production in conscious dogs challenged with either met-enkephalin or morphine (Konturek et al., 1980). Moreover, there is also evidence of *in vitro* acetylcholine release from cholinergic nerve endings in gastrointestinal tissue (ileal tissue, Paton, 1957). Acetylcholine also affects the secretion of pancreatic hormones, stimulating somatostatin release from human δ-islet cells via M1 muscarinic receptors (Molina et al., 2014). It remains unclear whether acetylcholine also stimulates ghrelin from gastrointestinal endocrine cells.

There is substantial evidence for cholinergic control of multiple gastrointestinal parameters, but it remains unclear whether ghrelin release is also under cholinergic control. There is evidence, albeit limited, for cholinergic stimulation of ghrelin release; fasting concentrations of ghrelin are decreased by the muscarinic antagonist, atropine (humans: Maier et al., 2004). Moreover, plasma concentrations of ghrelin are decreased in choline acetyltransferase-knockout neonatal mice (Lecomte et al., 2018). Moreover, acetylcholine evoked a small increase in ghrelin release from rat stomach tissue *in vitro* (Shrestha et al., 2009). There is, however, no information on cholinergic effects on ghrelin release or synthesis in the crop, proventriculus, and duodenum.

The present study examines the following: 1. ghrelin release and the expressions of *GHRL*, *GOAT*, and *GHSR-1a* in the crop, proventriculus, and the glandular equivalent of the mammalian stomach, along with the duodenum of newly hatched chicks; and 2*. in vitro* effects of cholinergic receptor antagonists on the ghrelin release and the expressions of *GHRL*, *GOAT*, and *GHSR-1a* in the crop, proventriculus, and duodenum of newly hatched chicks. The present study also compared the ghrelin parameters in newly hatched chicks and 1-day-old chicks. The rationale for using chicks of these ages was that the gastrointestinal tract would be more fully developed and putative confounding effects of physiological stress from hatching would be less likely in day 1 chicks than in day 0 chicks. Moreover, it is recognized that the ability of chicken ghrelin to stimulate contraction of the proventriculus is the maximum in newly hatched chicks (Kitazawa et al., 2013). Another rationale for using 0- and 1-day-old chicks without access to feed was to negate any confounding effects of ingesta on gastrointestinal functioning.

## 2 Materials and methods

### 2.1 Eggs and their incubation

Hatching eggs [mean egg weight = 60.3 ± 1.11 g] of the Ross 308 broiler chicken parental line (Aviagen) were obtained from a commercial farm in Poland. The eggs were incubated in a Brinsea-type OVA-Easy Advance Incubator under standard conditions, *i.e.*, a temperature of 37.8°C ± 0.1°C and a relative humidity (RH) of 50% ± 2%. Immediately after hatching (day 0) or 24 h later (day 1), chicks were transported to the laboratory and were euthanized within 2 h by cervical dislocation. Day 1 chicks had access to water. Feed was not provided to prevent confounding effects of ingesta influencing gastrointestinal parameters.

### 2.2 Animals

The experimental and animal procedures used in this study were performed in accordance with Directive 2010/63/EU of the European Parliament and the Council on the protection of animals used for scientific purposes. The animal study was reviewed and approved by the Institutional Animal Care and Use Committee at the Agricultural University in Krakow.

Experiment 1 was carried out on 20 newly hatched chicks (mixed sex) divided into four treatment groups (day 0); tissues were incubated *in vitro* without supplementation (0), with 100 nM of atropine (A), with hexamethonium (H), or with a combination of atropine and hexamethonium (A + H). Experiment 2 was carried out on 20 chicks (mixed sex) after 24 h of hatching that were divided into four treatment groups (day 1). The tissues were treated in the same manner as described in experiment 1.

### 2.3 Tissue culture

Fragments of crop, duodenum, and proventriculus (each fragment 50–70 mg) were dissected and placed on a 24-well plate. Tissues were incubated in 1 mL of Eagle’s medium supplemented with 0.05% bovine serum albumin and 2 µL of the antibiotic–antimycotic solution (AAS) (*n* = 5) for 6 h at 38°C (5% CO_2_) in the presence or absence of atropine, hexamethonium, and atropine with hexamethonium (100 nM). Doses of atropine and hexamethonium were determined during the pilot study: *in vitro* dose (1, 10, 100, and 1000 nM) responses and time (4, 5, and 6 h) responses of different tissues were taken from newly hatched chickens. The dose of 100 nM (100 mmol/L) and 6 h of culture time were chosen for the crop, duodenum, and proventriculus when the highest level of secretion was observed. The calculation was 1 nmol per 50 mg of tissue, which corresponds to 28.93 ng/mL medium/50 mg of tissue for atropine, and 36.21 ng/mL medium/50 mg of tissue for hexamethonium. Following incubation, the tissues were placed in StayRNA (A&A Biotechnology, Gdynia, Poland) until RNA isolation. The culture media were stored at −80^o^C for ghrelin determination.

### 2.4 Concentrations of hormones

Concentrations of total ghrelin (both acylated and deacylated forms) in the culture media were estimated using the radioimmunoassay kit, following the manufacturer’s protocol (DRG, Germany, RIA-3967). The assay parameters were evaluated in two ways:

1. standardization of ED 80, ED 50, and ED 20 with plasma chicken, sheep, and rat (each volume of 100, 300, and 500 µL); and 2. Evaluation of antibody binding of chicken standards at the concentrations of 50, 100, and 500 pg/mL. The final concentration of ghrelin was recalculated using the standardized parameters. There was close parallelism between the standard curve of mammalian ghrelin and dilutions of chicken plasma and low intra- and inter-assay coefficients of variance. The cross-reactivity was observed despite the marked differences between chicken ghrelin and that of mammals, other birds, and reptiles (see Supplementary Table S1). According to the manufacturer (DRG, Germany, RIA-3967), both acylated and deacylated ghrelin are equipotent in the assay, while the fragment ghrelin peptide (1–10) was non-detectable. This does not preclude that the epitope(s) detected in the assay include part of the N-terminal sequence (see Supplementary Table S1).

### 2.5 Gene expression analysis (RNA isolation, reverse transcription reaction, and qPCR reaction)

RNA was isolated with the TRI-Reagent according to the method described by Chomczynski and Sacchi (1987). The quality and concentration of the isolated RNA were determined by spectrophotometric analysis at wavelengths of 260 and 280 nm. Reverse transcription reactions were performed in accordance with the manufacturer’s recommendation. The reaction mixture contained 4.2 µL of sterile water, 2 µL of 10*×* RT buffer, 0.8 µL of 25*×* dNTP MIX (100 nM), 2 µL of 10*×* RT primer (random primer), 1 µL of MultiScribe^TM^ reverse transcriptase, and 2 µg of total RNA in 10 µL of water. Reverse transcription reactions were performed using a thermocycler (Personal Thermal Cycler, Eppendorf, Germany) in the cycle 25°C–10 min, 37°C–120 min, and 85°C–5 min. The obtained cDNA, stored at *−*20°C, constituted a template for the qPCR reaction . The qPCR reactions were performed in a 96-well thermal cycler (StepOnePlus, Applied Biosystems, Foster City, CA, United States). The 18S rRNA gene was used as a reference gene. The following program was used: 15 min at 95°C, followed by 40 cycles of 15 s at 95°C, 20 s at 62°C, and 20 s at 72°C; the reaction was performed in a 10-µL reaction mixture containing 2 µL of 5*×* Hot FIREPol EvaGreen qPCR mix, 0.12 µL of primers (10 pmol/μL), and 1 µL of cDNA (a 10-fold diluted sample from the RT reaction). A duplicate was performed for each sample. The relative number of genes analyzed was calculated by normalizing to the 18S rRNA reference gene (see Table 1). The expression was calculated using the 2^−^ΔΔ^Ct^ method. The StepOne program was used for quantification.

**TABLE 1:** Primers for ghrelin, GOAT, GHSR, and 18S rRNA used in the real-time PCR and amplified fragment size (bp).

Primer	F/R	Gene sequence	Fragment size (bp)
Ghrelin	F R	5′-GAAACTGCTCCCCTGGCTGGCTCTAG-3′ 5′-GAAGACAGACAGGCGATGTGTGG-3′	106
GOAT	F R	5′-GCTCTTCAGACTGGCGTACTACTCG-3′ 5′-GAAGACAGACAGGCGATGTGTGG-3′	145
GHSR	F R	5′-TCTTTTTCCTGCCCGTATTCTGC-3′ 5′-AGTCTGCTTGTTGTTCTTGTCCCTG-3′	125
18S rRNA	F R	5′-CTTTGGTCGCTCGCTCCTC-3′ 5′-CTGACCGGGTTGGTTTTGAT-3′	115

### 2.6 Reagents used in the research

The following reagents were used: Eagle’s medium (Biomed, Lublin, Poland), BSA and AAS (Merck KGaA, Darmstadt, Germany), StayRNA (A&A Biotechnology, Gdynia, Poland), TRI-Reagent (MRC Inc., Cincinnati, OH, United States), high-capacity cDNA reverse transcription kit (Thermo Fisher Scientific, Waltham, MA, United States), primers (IBB PAN, Warsaw, Poland), and 5× HOT FIREPol EvaGreen qPCR Mix Plus (ROX) (Solis BioDyne, Tartu, Estonia). Other reagents were purchased from Chempur (Piekary Slaskie, Poland), Warchem (Marki, Poland), and Sigma-Aldrich (St. Louis, MO, United States).

### 2.7 Statistical analysis

Data were analyzed using two-way analysis of variance (two-way ANOVA), *i.e.*, plus or minus atropine (a muscarinic cholinergic antagonist) and/or hexamethonium (a nicotinic cholinergic antagonist). Means were separated using Tukey’s test. Relationships between parameters were analyzed by linear regression.

## 3 Results

### 3.1 Differences by organ and day

The ghrelin release level was lower in the proventriculus than that in the crop and duodenum on day 0 but higher on day 1 (Table 2). The expressions of *GHRL*, *GOAT*, and *GHSR-1a* were similar in the crop, proventriculus, and duodenum on day 0. However, the expression of *GHRL* was much higher in the crop on day 1. The expression of *GOAT* was much lower in the proventriculus on day 1, while the expression of GHSR was lower in the proventriculus and duodenum on day 1.

**TABLE 2:** Ghrelin release and expression of ghrelin, GOAT, and GHSR by organ and age. Data are shown as the mean ± SEM.

	Ghrelin release mean ± SEM, pg mg^−1^, n = 5	Expression mean RQ relative to 18S rRNA ±SEM (n = 3)		
		Ghrelin	GOAT	GHSR
Day 0				
Crop	180.8 ± 2.1^a^	1.04 ± 0.029	1.26 ± 0.080^a^	1.01 ± 0.067
Proventriculus	41.4 ± 1.1^b^	1.20 ± 0.091	1.03 ± 0.070^a^	1.15 ± 0.047
Duodenum	141.8 ± 1.5^c^	1.05 ± 0.105	3.54 ± 0.372^b^	1.22 ± 0.121
Day 1				
Crop	155.6 ± 2.1	108.5 ± 0.538^b^	33.8 ± 1.94^b^	108.5 ± 0.54^b^
Proventriculus	239.4 ± (5) 2.9	0.287 ± 0.023^a^	0.797 ± 0.033^a^	0.583 ± 0.028^a^
Duodenum	132.8 ± (5) 1.6	0.670 ± 0.082^a^	35.5 ± 0.372^b^	1.49 ± 0.043^a^

^a,b,c^ Different superscript letters indicate difference between organs *P* < 0.05.

### 3.2 Effects of cholinergic antagonists

**FIGURE 1:**
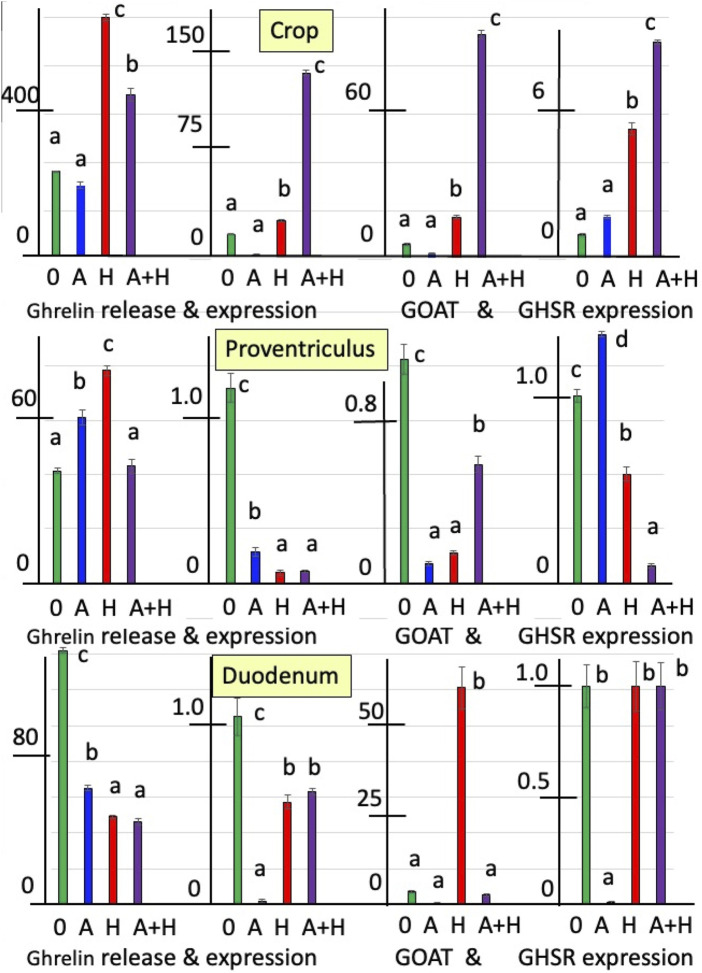
*In vitro* effects of atropine (A) and/or hexamethonium (H) on ghrelin release (pg mg^-1^ 6 h^-1^) and expression of ghrelin-related genes (*GHRL*, *GOAT,* and *GHSR-1a* shown relative to control as 1.0) from explants of the crop, proventriculus, and duodenum from newly hatched (day-0) chicks. ^a, b, c,d^ Different superscript letters indicate difference at *P* < 0.05.

**FIGURE 2:**
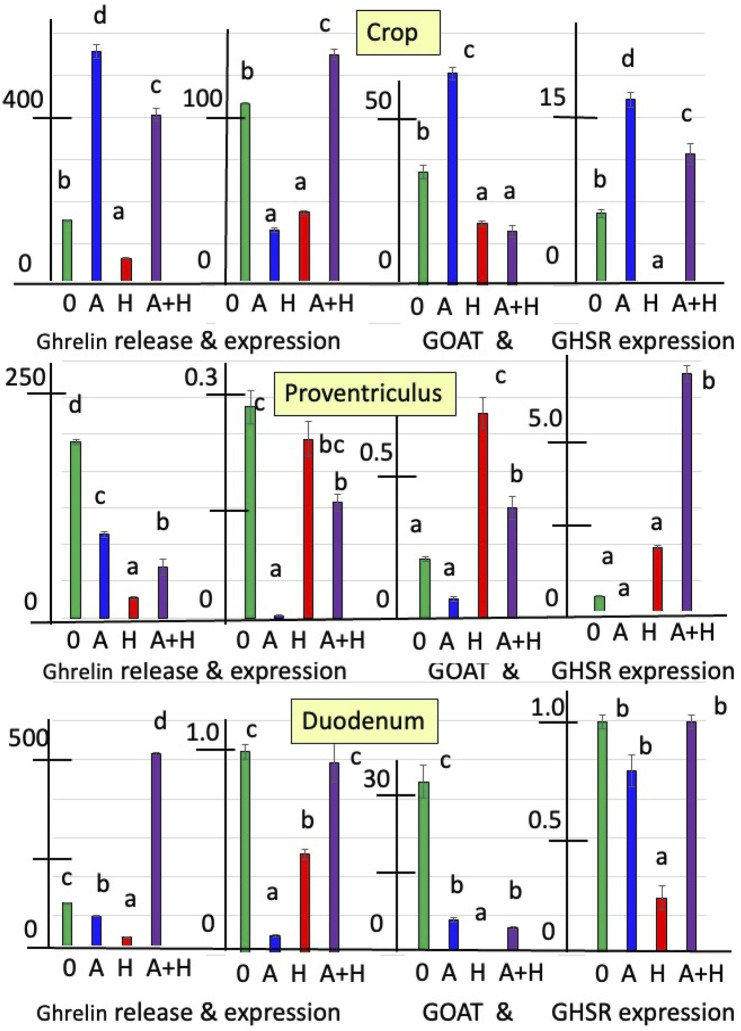
*In vitro* effects of atropine (A) and/or hexamethonium (H) on ghrelin release (pg mg^−1^ 6 h^−1^) and expression of ghrelin-related genes (*GHRL, GOAT*, and *GHSR-1a* shown relative to control as 1.0 of day 0) from explants of the crop, proventriculus, and duodenum from hatched (day-1) chicks. ^a, b, c^ Different superscript letters indicate difference at *P* < 0.05.

Figures 1, 2 summarize the effects of cholinergic antagonists (atropine and hexamethonium) on the release of ghrelin, together with the expressions of *GHRL*, *GOAT*, and *GHSR* in explants of the chick crop, proventriculus, and duodenum incubated *in vitro* for 6 h.

In newly hatched chicks (day-0 chicks), the release of ghrelin was increased (*P* < 0.001) by 2.76-fold from crop explants and by 89.4% from proventriculus explants in the presence of the nicotinic cholinergic antagonist, hexamethonium (Figure 1; Supplementary Table S2). In contrast, the release of ghrelin from the duodenum was decreased (*P* < 0.001) in the presence of hexamethonium by 65.4% (Figure 1; Supplementary Table S2). In addition, in the presence of atropine, the release of ghrelin was increased (*P* < 0.001) in proventriculus explants (by 47.8%) but decreased (*P* < 0.001) in duodenal explants (by 54.1%) (Figure 1; Supplementary Table S2). There were interactions (*P* < 0.0002) between the effects of atropine and hexamethonium (Supplementary Table S2). The *in vitro* release of ghrelin from crop and proventriculus explants was decreased in the presence of hexamethonium alone (Figure 1; Supplementary Table S2).

The expression of *GHRL* in explants incubated *in vitro* with hexamethonium was increased in crop tissue (by 25.5-fold) but decreased in proventriculus (by 94.7%) and duodenal tissue (by 45.4%) (Figure 1; Supplementary Table S3). *GHRL* expression was also decreased in the presence of atropine in the crop (by 78.9%), proventriculus (by 84.1%), and duodenum tissues (by 98.0%) (Figure 1; Supplementary Table S3). There were interactions (*P* < 0.0001) between the effects of atropine and hexamethonium (Table 3 and Figure 1; Supplementary Table S3). In crop explants incubated with both atropine and hexamethonium, *GHRL* expression was markedly increased, being 582.3-fold greater than that with atropine alone and 4.83-fold greater than that with hexamethonium alone (Figure 1; Supplementary Table S3). In contrast, the expression of *GHRL* from either proventriculus or duodenal explants in the presence of both atropine and hexamethonium was not different (*P* > 0.05) from that in the presence of hexamethonium alone (Figure 1; Supplementary Table S3).

**TABLE 3:** Relationships/regressions [adjusted R^2^ (*P* <)] between ghrelin-related parameters.

	Ghrelin release	Ghrelin expression	GOAT expression	GHSR expression
**Crop**				
**Day 0**				
Ghrelin release	1.00	0.268 (*P* < 0.05)	0.282 (*P* < 0.05)	−0.009
Ghrelin expression		1.00	0.995 (*P* < 0.001)	0.861 (*P* < 0.001)
GOAT expression			1.00	0.841
GHSR expression				1.00
**Day 1**				
Ghrelin release	1.00	0.716 (*P* < 0.001)	0.188	−0.022
Ghrelin expression		1.00	0.238	−0.098
GOAT expression			1.00	0.387 (*P* < 0.05)
GHSR expression				1.00
**Proventriculus**				
**Day 0**				
Ghrelin release	1.00	0.209	0.638 (*P* < 0.001)	−0.082
Ghrelin expression		1.00	0.641 (*P* < 0.001)	0.638 (*P* < 0.001)
GOAT expression			1.00	0.331 (*P* < 0.05)
GHSR expression				1.00
**Day 1**				
Ghrelin release	1.00	−0.078	0.478 (*P* < 0.01)	0.173
Ghrelin expression		1.00	0.161	−0.096
GOAT expression			1.00	0.032
GHSR expression				1.00
**Duodenum**				
**Day 0**				
Ghrelin release	1.00	−0.029	0.080	−0.037
Ghrelin expression		1.00	−0.098	0.645 (*P* < 0.001)
GOAT expression			1.00	0.040
GHSR expression				1.00
**Day 1**				
Ghrelin release	1.00	0.217	−0.085	0.271 (*P* < 0.05)
Ghrelin expression		1.00	0.202	0.119
GOAT expression			1.00	0.239
GHSR expression				1.00

**Bold** indicates P < 0.05.

Cholinergic antagonists affected the expression of *GOAT*. *GOAT* expression was markedly increased (*P* < 0.0001) in either crop (13.9-fold) or duodenal tissue (17.1-fold) incubated with hexamethonium (Figure 1; Supplementary Table S4). In contrast, the expression of *GOAT* was decreased (*P* < 0.001) in either proventriculus or duodenal tissue incubated with atropine (Figure 1; Supplementary Table S4). There were interactions (*P* < 0.0001) between the effects of atropine and hexamethonium (Figure 1; Supplementary Table S4). For instance, the expression of ghrelin in the presence of atropine and hexamethonium was greater than that in the presence of either atropine (185.4-fold) or hexamethonium (6.0-fold) alone in crop tissues (Figure 1; Supplementary Table S4). Similarly, the depressive effects of either atropine or hexamethonium on *GOAT* expression in proventriculus explants were ameliorated in the presence of atropine and hexamethonium (Figure 1; Supplementary Table S4). Furthermore, the stimulatory effect of hexamethonium on *GOAT* expression in the duodenum was decreased (*P* < 0.0001; by 95.4%) in the presence of atropine (Figure 1; Supplementary Table S4).

The expression of *GHSR-1a* was influenced by cholinergic antagonists. Expression in crop tissue was increased 5.33-fold (*P* < 0.0001) in the presence of hexamethonium (Figure 1; Supplementary Table S2). Expression in proventriculus tissue was increased by 33.0% (*P* < 0.01) in the presence of atropine but decreased by 41.7% (*P* < 0.001) in the presence of hexamethonium (Figure 1; Supplementary Table S4). The expression of *GHSR-1a* in duodenal explants was decreased (*P* < 0.0001) by 99.1% when incubated in the presence of atropine (Figure 1; Supplementary Table S4). There were interactions (*P* < 0.0001) between the effects of atropine and hexamethonium (Figure 1; Supplementary Table S4). Atropine increased (*P* < 0.01) hexamethonium stimulation of *GHSR-1a* expression in crop explants but decreased (*P* < 0.0001) GHSR expression in proventriculus explants (Figure 1; Supplementary Table S4). Moreover, in duodenal explants, in the presence of both hexamethonium and atropine, the inhibitory effects of atropine were lost (Figure 1; Supplementary Table S4).

For day-1 chicks, there was increased release of ghrelin from crop explants incubated with atropine (by 3.58-fold) (*P* < 0.0001) (Figure 2; Supplementary Table S2). In contrast, hexamethonium decreased ghrelin release (*P* < 0.001) (Figure 2; Supplementary Table S2). However, ghrelin release from either proventriculus or duodenal explants was decreased (*P* < 0.001) in the presence of atropine (proventriculus: 52.5% and duodenum: 25.6%) or hexamethonium (proventriculus: 88.8% and duodenum: 65.5%) (Figure 2; Supplementary Table S2). There were interactions (*P* < 0.01) between the effects of atropine and hexamethonium (Figure 2; Supplementary Table S2). Hexamethonium decreased (*P* < 0.01) atropine-stimulated ghrelin release from crop explants (Figure 2; Supplementary Table S2). Although atropine or hexamethonium alone decreased the release of ghrelin, there was increased (*P* < 0.0001) (by 3.88-fold) ghrelin release in the presence of both cholinergic antagonists compared to the control (zero additions) in duodenal explants (Figure 2; Supplementary Table S2).

*GHRL* expression was decreased in crop tissue incubated with either atropine (by 69.5% %) or hexamethonium (by 59.7% %) (Figure 2; Supplementary Table S3). In the presence of atropine, there was suppression (*P* < 0.001) of ghrelin expression from proventriculus (by 99.0%) or duodenal explants (by 90.9%) (Figure 2; Supplementary Table S3). There were interactions (*P* < 0.001) between the effects of atropine and hexamethonium. Atropine and hexamethonium together stimulated (*P* < 0.01) ghrelin expression in crop explants. This was in contrast to the inhibitory effects observed when each antagonist was used alone (Figure 2; Supplementary Table S3).

The expression of *GOAT* in crop explants was increased (*P* < 0.001) in the presence of atropine (by 86.7%) but inhibited by hexamethonium (by 45.3%) (Figure 2; Supplementary Table S4). The situation was reversed in proventriculus explants, where atropine inhibited *GOAT* expression by 67.8% (*P* < 0.01), while hexamethonium stimulated it by 3.50-fold (*P* < 0.0001) (Figure 2; Supplementary Table S4). Both cholinergic antagonists reduced (*P* < 0.0001) GOAT expression in the duodenum, 79.9% for atropine and 99.9% for hexamethonium (Figure 2; Supplementary Table S4).

*GHSR-1a* in crop explants was increased (*P* < 0.001) by incubation with atropine (by 156.5%) and decreased (P < 0.001) by incubation with hexamethonium (by 94.9%) (Figure 2; Supplementary Table S5). Atropine decreased (*P* < 0.01) the expression of *GHSR-1a* (by 70.8%), while incubation with hexamethonium increased (*P* < 0.001) *GHSR-1a* expression in proventriculus explants (by 3.29-fold) (Figure 2; Supplementary Table 5). There was decreased (*P* < 0.001) expression of *GHSR-1a* in duodenal explants incubated with hexamethonium (Figure 2; Supplementary Table S5). There were interactions between the effects of atropine and hexamethonium. For instance, the expression of *GHSR-1a* was increased in the presence of both atropine and hexamethonium in both crop and proventriculus explants (Figure 2; Supplementary Table S5).

### 3.3 Relationships between ghrelin release and *GHRL* expression, along with the expressions of *GOAT* and *GHSR* in tissues incubated in the presence or absence of atropine and/or hexamethonium

In crop explants from day-0 chicks incubated with or without atropine and/or hexamethonium, there were very strong relationships (adjusted R^2^ > 0.84) between the expression of ghrelin and that of *GOAT* (*P* = 2.97 E^-13^; slope 0.721 ± 0.014) and that of *GHSR-1a* (*P* = 8.23E^-6^; slope 0.057 ± 0.007), as well as between the expressions of *GOAT* and *GHSR-1a* (*P* = 1.65 E^-5^; slope 0.077 ± 0.010) (Table 3). There were also relationships (*P* < 0.05) between ghrelin release and *GHRL* expression (*P* = 0.049; slope −0.462 ± 0.206) and between ghrelin release and *GOAT* expression (*P* = 0.044; slope −0.340 ± 0.148).

There were a series of relationships (*P* < 0.05) between ghrelin-related parameters in proventriculus explants in day-0 chicks (Table 3). Relationships (*P* < 0.05) were observed between ghrelin release and *GOAT* expression (*P =* 0.001; slope −0.020 ± 0.004), between ghrelin expression and *GOAT* expression (*P <* 0.001; slope 0.655 ± 0.144), between ghrelin expression and *GHSR-1a* expression (*P <* 0.001; slope 0.731 ± 0.162), and between the expressions of GOAT and GHSR (*P* = 0.029; slope 0.699 ± 0.275) (Table 3). In contrast, there was only one relationship between ghrelin-related parameters in proventriculus explants from day 1-chicks, i.e., between ghrelin release and *GOAT* expression (*P* = 0.0076; slope −0.0088 ± 0.0026) (Table 3).

There were only two relationships between ghrelin-related parameters in duodenal explants from day-0 and -1 chicks, i.e., between the expressions of *GHRL* and *GHSR-1a* (day 0: *P <* 0.001; slope 1.20 ± 0.26) and between ghrelin release and *GHSR-1a* expression (day 1: *P* = 0.047; slope 0.00147 ± 0.0007) (Table 3).

## 4 Discussion

In the present study, ghrelin was released *in vitro* from crop explants, along with the explants of proventriculus and duodenal tissue from newly hatched chicks (Table 2). In addition, *GHRL* and *GOAT* were expressed in these tissues (Table 2). Although these observations are novel, there is evidence of ghrelin’s role in the gastrointestinal tract, with ghrelin influencing the motility of the gastrointestinal tract in chickens (reviewed by Kitazawa and Kaiya, 2019). For instance, ghrelin stimulates contractions of chicken crop strips *in vitro* (Kitazawa et al., 2007). The present observations of *GHSR-1a* expression in the crop and proventriculus (Table 2) are consistent with the report of *GHSR-1a* in the muscle of chicken crop and both the smooth muscle and enteric neurons in the proventriculus (Kitazawa et al., 2007). The presence of ghrelin release, along with the expressions of *GHRL*, *GOAT*, and *GHSR-1a* in the proventriculus of chicks, is analogous to their presence in the mammalian stomach (Kojima et al., 1999; Date et al., 2000; Ariyasu et al., 2001). However, there is no parallel to the presence of a crop in chickens in mammals as mammals do not have a crop. The occurrence of ghrelin release, along with the expressions of *GHRL*, *GOAT*, and *GHSR-1a* in the chick duodenum, is similar to that observed in mammals (Date et al., 2000; Ariyasu et al., 2001).

Both muscarinic and nicotinic antagonists affect ghrelin release and the expressions of *GHRL*, GOAT, and *GHSR-1a* (Figures 1, 2) in explants of the chick crop, proventriculus, and duodenum tissues. Similarly, acetylcholine stimulates somatostatin release from human δ-islet cells via M1 muscarinic receptors (Molina et al., 2014). Moreover, there is substantial evidence that acetylcholine or cholinergic agonists increase insulin release from pancreatic β-islet cells, probably via M3 muscarinic receptors (rats, Boschero et al., 1995; mice, Duttaroy et al., 2004; Zawalich et al., 2004; Gautam et al., 2006; human, Molina et al., 2014). It was noted that α-pancreatic islet cells release acetylcholine with the muscarinic antagonist, atropine, blocking acetylcholine release (Rodriguez-Diaz et al., 2011). Hence, the source of acetylcholine stimulating insulin may be either neuronal or the α-islet cells. In contrast, no changes in glucagon release were observed in human pancreatic islet cells in the presence of either acetylcholine or cholinergic agonists (Molina et al., 2014).

There were strong relationships (*P* < 0.01) between the expressions of *GHRL* and *GOAT* (adjusted R^2^ for crop: 0.996 and for proventriculus: 0.641), expressions of ghrelin and GHSR (adjusted R^2^ for the crop: 0.861 and for the proventriculus: 0.638), and expressions of *GOAT* and *GHSR-1a* (adjusted R^2^ for crop: 0.841 and for the proventriculus: 0.331) in tissue from day-0 chicks within 2 h of hatching (Table 3). Relationships were either weak or non-existent with duodenal tissue or tissue from 1-day-old chicks. These data support a tight tie between the development of ghrelin and related parameters in the crop and proventriculus.

There is evidence of cross talk between gastrointestinal peptides and the cholinergic system, with atropine blocking the effect of motilin on contractions of the chicken small intestine *in vitro* (Kitazawa et al., 1995). Atropine inhibits ghrelin-stimulated contractions of chicken proventriculus strips *in vitro* (Kitazawa et al., 2007).

**FIGURE 3:**
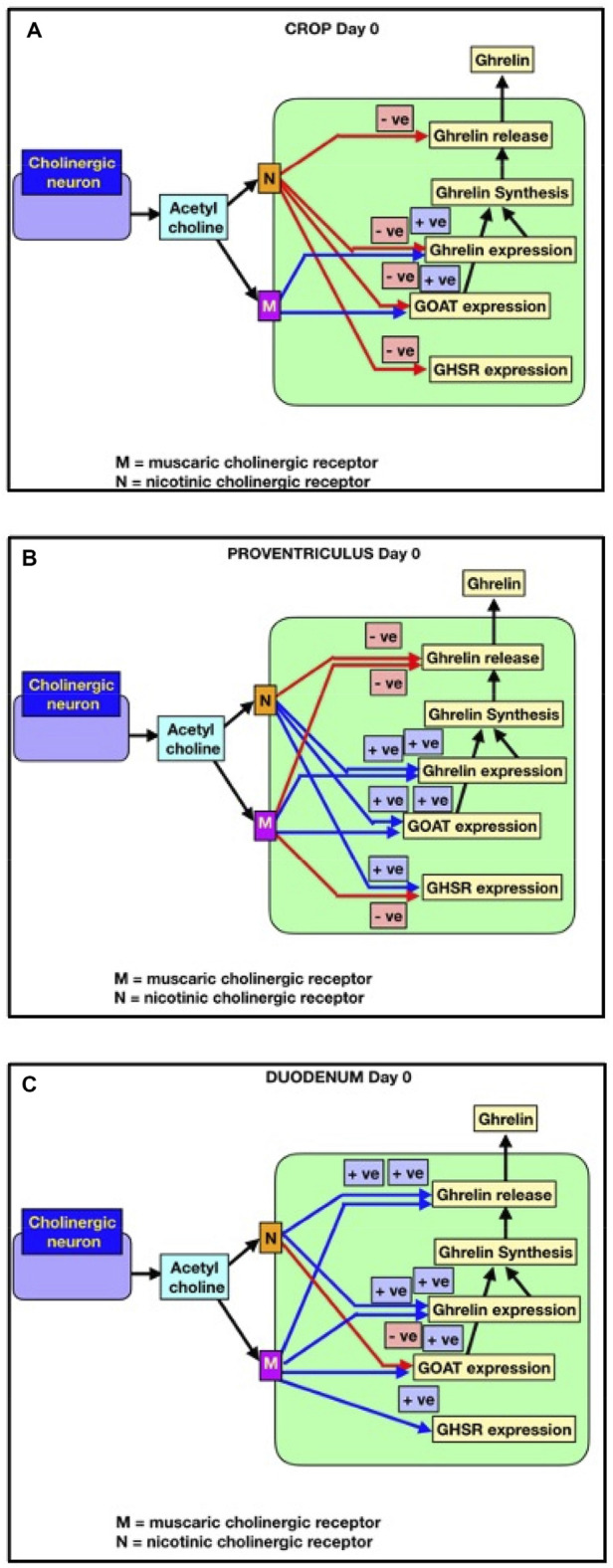
Cholinergic control of ghrelin release and synthesis (*GHRL* expression), together with *GHSR-1a* and *GOAT* in newly hatched chicks (within 2 hours of hatching). Red lines indicate negative effects. Blue lines indicate positive effects. **(A)** Crop; **(B)** proventriculus; **(C)** duodenum.

The effects of cholinergic antagonists in the present study are consistent with the tonic release of acetylcholine from nerve endings or other cells. There is evidence of *in vitro* acetylcholine release from cholinergic nerve endings in gastrointestinal tissue (ileal tissue: Paton, 1957). It remains unclear whether the acetylcholine influencing ghrelin parameters originates from cholinergic nerve endings or acetylcholine-producing cells, in a manner similar to its release from pancreatic islet cells (Rodriguez-Diaz et al., 2011). The present results on the effects of cholinergic antagonists are consistent with the models (Figure 3). It is clear that within an age, there were consistent increases in all ghrelin-related parameters in crop explants from newly hatched chicks incubated with hexamethonium. However, there are consistent decreases in all ghrelin-related parameters in crop explants from day-1 chicks incubated with hexamethonium and with proventriculus and duodenal explants from day-1 chicks incubated with atropine. There are also marked differences in the effect of cholinergic antagonists between newly hatched chicks (day 0) and day-1 chicks. The explanation for this remains unclear. However, the marked differences in the effect of cholinergic receptor antagonists between newly hatched chicks (day 0) and day-1 chicks support the hypothesis that acetylcholine (synthesized by gastrointestinal cells and/or released from the vagus nerve) controls ghrelin release and synthesis during avian gastrointestinal growth and development.

## 5 Conclusion

Ghrelin is synthesized in and released from explants of the crop, proventriculus, and duodenum of newly hatched and 1-day-old chicks. There are strong relationships between the expressions of *GHRL*, *GOAT*, and *GHSR-1a* in both the crop and duodenum from newly hatched chicks. Cholinergic antagonists influenced both ghrelin release and synthesis in the crop, proventriculus, and duodenum, supporting the presence of cholinergic control.

## Data Availability

The datasets presented in this study can be found in online repositories. The names of the repository/repositories and accession number(s) can be found in the article/Supplementary Material.
