# Supplementary material for: Effects of cholinergic antagonists on ghrelin release and expression in the crop, proventriculus, and duodenum of newly hatched chickens
Source: Front Physiol. 2025 May 2;16:1553474. doi: 10.3389/fphys.2025.1553474 (PMC12082714; doi:10.3389/fphys.2025.1553474)
Supplement: Supplementary file 1 [file DataSheet1.docx]

**SUPPLEMENTARY TABLE 1.** *In vitro* Effects on Atropine and/or Hexamethonium on Ghrelin Release from Explants of Crop, Proventriculus and Duodenum from Newly Hatched Chicks

|  | Mean release of ghrelin + (n = 5) SEM pg mg^-1^ | |
| --- | --- | --- |
|  | **Day 0** | **Day 1** |
| **Crop** |  |  |
| Control | 180.8 + 2.1^a^ | 155.6 + 2.1^b^ |
| Atropine | 153.0 + 6.4^a^ | 557.1 + 15.2^d^ |
| Hexamethonium | 498.4 + 6.8^c^ | 66.0 + 0.8^a^ |
| Atropine + Hexamethonium | 339.7 + 14.0^b^ | 407.3 + 15.2^c^ |
| **2 way ANOVA** | *P* = | *P* = |
| Atropine | 4.15E^-9^ | 1.85E^-10^ |
| Hexamethonium | 8.25E^-6^ | 1.56E^-6^ |
| Interaction | 0.000132 | 0.00946 |
|  |  |  |
| **Proventriculus** |  |  |
| Control | 41.4 + 1.1^a^ | 239.4 + 2.9^a^ |
| Atropine | 61.2 + 2.75^b^ | 113.6 + 3.9^b^ |
| Hexamethonium | 78.4 + 1.75^c^ | 26.8 + 1.50^d^ |
| Atropine + Hexamethonium | 43.0 + 2.42^a^ | 69.2 + 10.7^c^ |
| **2 way ANOVA** | *P* = | *P* = |
| Atropine | 0.000275 | 7.19E^-12^ |
| Hexamethonium | 4.00E^-5^ | 9.57E^-22^ |
| Interaction | 1.73E^-11^ | 1.55E^-17^ |
|  |  |  |
| **Duodenum** |  |  |
| Control | 141.8 + 1.5^a^ | 132.8 + 1.6^a^ |
| Atropine | 65.0 + 1.6^b^ | 98.8 + 1.6^b^ |
| Hexamethonium | 49.0 + 1.14^c^ | 45.8 + 0.9^c^ |
| Atropine + Hexamethonium | 46.2 + 1.72^c^ | 515.6 + 2.8^d^ |
| **2 way ANOVA** | *P* = | *P* = |
| Atropine | 5.41E^-17^ | 5.23E^-22^ |
| Hexamethonium | 3.24E^-14^ | 2.56E^-19^ |
| Interaction | 1.12E^-14^ | 2.72E^-26^ |

^a, b, c, d^ Different superscript letters indicate difference between treatments *P* < 0.05

**SUPPLEMENTARY TABLE 2.** *In vitro* Effects on Atropine and/or Hexamethonium on Ghrelin Expression in Explants of Crop, Proventriculus and Duodenum from Newly Hatched Chicks

|  | Ghrelin expression  mean RQ relative to 18S rRNA + (n = 3) SEM | |
| --- | --- | --- |
|  | **Day 0** | **Day 1** |
| **Crop** |  |  |
| Control | 1.04 **+** 0.029^a^ | 108.5 **+** 0.54^a^ |
| Atropine | 0.22 **+** 0.017^a^ | 33.1 + 1.67^b^ |
| Hexamethonium | 26.51 **+** 0.80^b^ | 43.7 + 0.90^b^ |
| Atropine + Hexamethonium | 128.1 + 1.82^c^ | 137.6 + 2.82^c^ |
| **2 way ANOVA** | *P =* | *P =* |
| Atropine | 2.57E^-11^ | 0.000652 |
| Hexamethonium | 8.98E^-13^ | 2.88E^-6^ |
| Interaction | 2.26E^-11^ | 3.18E^-11^ |
|  |  |  |
| **Proventriculus** |  |  |
| Control | 1.20 **+** 0.091^a^ | 0.287 **+** 0.023^a^ |
| Atropine | 0.19 + 0.029^c^ | 0.003 + 0.001^c^ |
| Hexamethonium | 0.063 + 0.016^b^ | 0.243 + 0.024^ab^ |
| Atropine + Hexamethonium | 0.070 + 0.0041^b^ | 0.157 + 0.012^b^ |
| **2 way ANOVA** | *P =* | *P =* |
| Atropine | 3.23E^-5^ | 6.28E^-6^ |
| Hexamethonium | 0.299 | 0.0145 |
| Interaction | 1.45E^-5^ | 0.000546 |
|  |  |  |
| **Duodenum** |  |  |
| Control | 1.05 **+** 0.105^a^ | 0.693 + 0.024^a^ |
| Atropine | 0.020 + 0.008^c^ | 0.063 + 0.004^c^ |
| Hexamethonium | 0.573 + 0.041^b^ | 0.343 + 0.019^b^ |
| Atropine + Hexamethonium | 0.630 + 0.020^b^ | 0.657 + 0.066^a^ |
| **2 way ANOVA** | *P =* | *P =* |
| Atropine | 3.88E^-6^ | 0.00249 |
| Hexamethonium | 0.0787 | 0.0103 |
| Interaction | 1.63E^-6^ | 1.23E^-6^ |

^a, b, c, d^ Different superscript letters indicate difference between treatments *P* < 0.05

**SUPPLEMENTARY TABLE 3.** *In vitro* Effects on Atropine and/or Hexamethonium on GOAT Expression in Explants of Crop, Proventriculus and Duodenum from Newly Hatched Chicks

|  | GOAT expression  mean RQ relative to 18S rRNA + (n = 3) SEM | |
| --- | --- | --- |
|  | **Day 0** | **Day 1** |
| **Crop** |  |  |
| Control | 1.26 **+** 0.080^a^ | 33.8 + 1.94^a^ |
| Atropine | 0.50 **+** 0.040^a^ | 63.1 + 1.98^b^ |
| Hexamethonium | 17.5 **+** 0.62^b^ | 18.5 + 0.79^c^ |
| Atropine + Hexamethonium | 92.7 **+** 1.87^c^ | 16.2 + 1.74^c^ |
| **2 way ANOVA** | ***P =*** | ***P =*** |
| Atropine | 2.59E^-10^ | 4.22E^-5^ |
| Hexamethonium | 1.32E^-11^ | 7.54E^-8^ |
| Interaction | 2.25E^-10^ | 1.35E^-5^ |
|  |  |  |
| **Proventriculus** |  |  |
| Control | 1.03 **+** 0.070^a^ | 0.797 + 0.033^a^ |
| Atropine | 0.087 **+** 0.009^c^ | 0.257 + 0.034^a^ |
| Hexamethonium | 0.137 **+** 0.012^c^ | 2.79 + 0.205^c^ |
| Atropine + Hexamethonium | 0.547 **+** 0.038^b^ | 1.50 + 0.153^b^ |
| **2 way ANOVA** | ***P =*** | ***P =*** |
| Atropine | 0.00019 | 0.000106 |
| Hexamethonium | 0.00075 | 1.62E^-6^ |
| Interaction | 1.84E^-7^ | 0.0196 |
|  |  |  |
| **Duodenum** |  |  |
| Control | 3.54 + 0.372^a^ | 35.5 +3.33^a^ |
| Atropine | 0.34 + 0.111^a^ | 7.13 + 0.516^b^ |
| Hexamethonium | 60.6 + 5.87^b^ | 0.01 + 0.00^c^ |
| Atropine + Hexamethonium | 2.76 + 0.301^a^ | 5.56 + 0.291^b^ |
| **2 way ANOVA** | ***P =*** | ***P =*** |
| Atropine | 6.48 E^-5^ | 0.000146 |
| Hexamethonium | 7.87E^-6^ | 4.299E^-6^ |
| Interaction | 1.48E^-5^ | 8.37E^-6^ |

^a, b, c, d^ Different superscript letters indicate difference between treatments *P* < 0.05

**SUPPLEMENTARY TABLE 4.** *In vitro* Effects on Atropine and/or Hexamethonium on GHSR-1a Expression in Explants of Crop, Proventriculus and Duodenum from Newly Hatched Chicks

|  | GHSR-1a expression  mean RQ relative to 18S rRNA + (n = 3) SEM | |
| --- | --- | --- |
|  | **Day 0** | **Day 1** |
| **Crop** |  |  |
| Control | 1.01 **+** 0.067^a^ | 6.90 **+** 0.384^a^ |
| Atropine | 1.75 **+** 0.081^a^ | 17.7 **+** 0.74^b^ |
| Hexamethonium | 5.38 + 0.393^b^ | 0.35 + 0.075^d^ |
| Atropine + Hexamethonium | 8.95 + 0.162^c^ | 12.5 + 0.97^c^ |
| **2 way ANOVA** | ***P =*** | ***P =*** |
| Atropine | 9.58E^-6^ | 1.281E^-9^ |
| Hexamethonium | 4.46E^-9^ | 2.44E^-7^ |
| Interaction | 0.000191 | 0.107 |
|  |  |  |
| **Proventriculus** |  |  |
| Control | 1.15 **+** 0.042^a^ | 0.583+ 0.028^a^ |
| Atropine | 1.53 **+** 0.015^d^ | 0.170 **+** 0.042^a^ |
| Hexamethonium | 0.67 + 0.046^b^ | 1.92**+** 0.099^a^ |
| Atropine + Hexamethonium | 0.103 + 0.015^c^ | 6.65 + 0.52^b^ |
| **2 way ANOVA** | ***P =*** | ***P =*** |
| Atropine | 1.04E^-8^ | 3.74E^-5^ |
| Hexamethonium | 3.88E^-5^ | 4.30E^-7^ |
| Interaction | 0.000168 | 1.03E^-5^ |
|  |  |  |
| **Duodenum** |  |  |
| Control | 1.22 **+** 0.121^a^ | 1.49 + 0.043^a^ |
| Atropine | 0.011 + 0.005^b^ | 1.18 + 0.101^a^ |
| Hexamethonium | 1.22 + 0.139^a^ | 0.363+ 0.076^b^ |
| Atropine + Hexamethonium | 1.22 + 0.133^a^ | 1.49+ 0.043^a^ |
| **2 way ANOVA** | ***P =*** | ***P =*** |
| Atropine | 0.00070 | 0.000391 |
| Hexamethonium | 0.00070 | 0.000391 |
| Interaction | 0.00070 | 7.62E^-6^ |

^a, b, c, d^ Different superscript letters indicate difference between treatments *P* < 0.05
